# Performance evaluation of indel calling tools using real short-read data

**DOI:** 10.1186/s40246-015-0042-2

**Published:** 2015-08-19

**Authors:** Mohammad Shabbir Hasan, Xiaowei Wu, Liqing Zhang

**Affiliations:** Department of Computer Science, Virginia Tech, Blacksburg, VA 24061 USA; Department of Statistics, Virginia Tech, Blacksburg, VA 24061 USA

**Keywords:** Indel calling, Variant calling, HaplotypeCaller, Next-generation sequencing, Deep sequencing, Software evaluation

## Abstract

**Background:**

Insertion and deletion (indel), a common form of genetic variation, has been shown to cause or contribute to human genetic diseases and cancer. With the advance of next-generation sequencing technology, many indel calling tools have been developed; however, evaluation and comparison of these tools using large-scale real data are still scant. Here we evaluated seven popular and publicly available indel calling tools, GATK Unified Genotyper, VarScan, Pindel, SAMtools, Dindel, GTAK HaplotypeCaller, and Platypus, using 78 human genome low-coverage data from the 1000 Genomes project.

**Results:**

Comparing indels called by these tools with a known set of indels, we found that Platypus outperforms other tools. In addition, a high percentage of known indels still remain undetected and the number of common indels called by all seven tools is very low.

**Conclusion:**

All these findings indicate the necessity of improving the existing tools or developing new algorithms to achieve reliable and consistent indel calling results.

**Electronic supplementary material:**

The online version of this article (doi:10.1186/s40246-015-0042-2) contains supplementary material, which is available to authorized users.

## Introduction

Insertion and deletion (indel), is a common form of polymorphism corresponding to the addition or removal of base pairs in the DNA sequence of an organism. Indels have been recognized as the second most abundant source of genetic variation in human populations [1–3]. Studies have shown that in the human body, 16 to 25 % of all sequence polymorphisms are indels [4]. Furthermore, indels have been identified to play a key role in causing diseases. For example, cystic fibrosis, a common genetic disease, is frequently caused by deletion of three nucleotides in the coding region of the CFTR gene [5]. Diseases such as fragile X syndrome [6], trinucleotide repeat disorders [7], Mendelian disorders [8], Bloom syndrome [9], acute myeloid leukemia [10–12], and lung cancer [13] are often caused by short repeats/insertions in the DNA sequence. Moreover, insertion of transposable elements such as Alu, L1, and SVA can interrupt gene function and cause diseases like hemophilia, neurofibromatosis, muscular dystrophy, and cancer [14]. In addition, indels can also change gene expression by altering phasing and spacing of DNA sequences in the promoter regions [15]. For example, a small insertion of 5 bps can rotate the binding site to the opposite face of the DNA helix, whereas a long insertion of 100 bps can increase the spacing between two binding sites [3]. Therefore, indels in the promoter regions might explain certain difference in gene expression observed in humans [15] and can be used as genetic markers in natural populations [16]. Since indels influence human traits and diseases, detection of indels in a reliable manner is a prerequisite to develop effective treatment and medicine [17, 18].

In recent time, next-generation sequencing (NGS) has become more convenient because of its high efficiency, improved sensitivity of different sequencing platforms, and reduced cost as compared to Sanger sequencing [19, 20]. By applying NGS in a large scale, whole genome sequencing (WGS) is now possible at an individual level [21–23] and it has revealed a significant number of structural variants that were not reported previously. Since indels can alter human traits and cause diseases, the result of indel calling from individual WGS can be used to predict the future health of sampled individuals and to develop customized medical treatments.

**Fig. 1:**
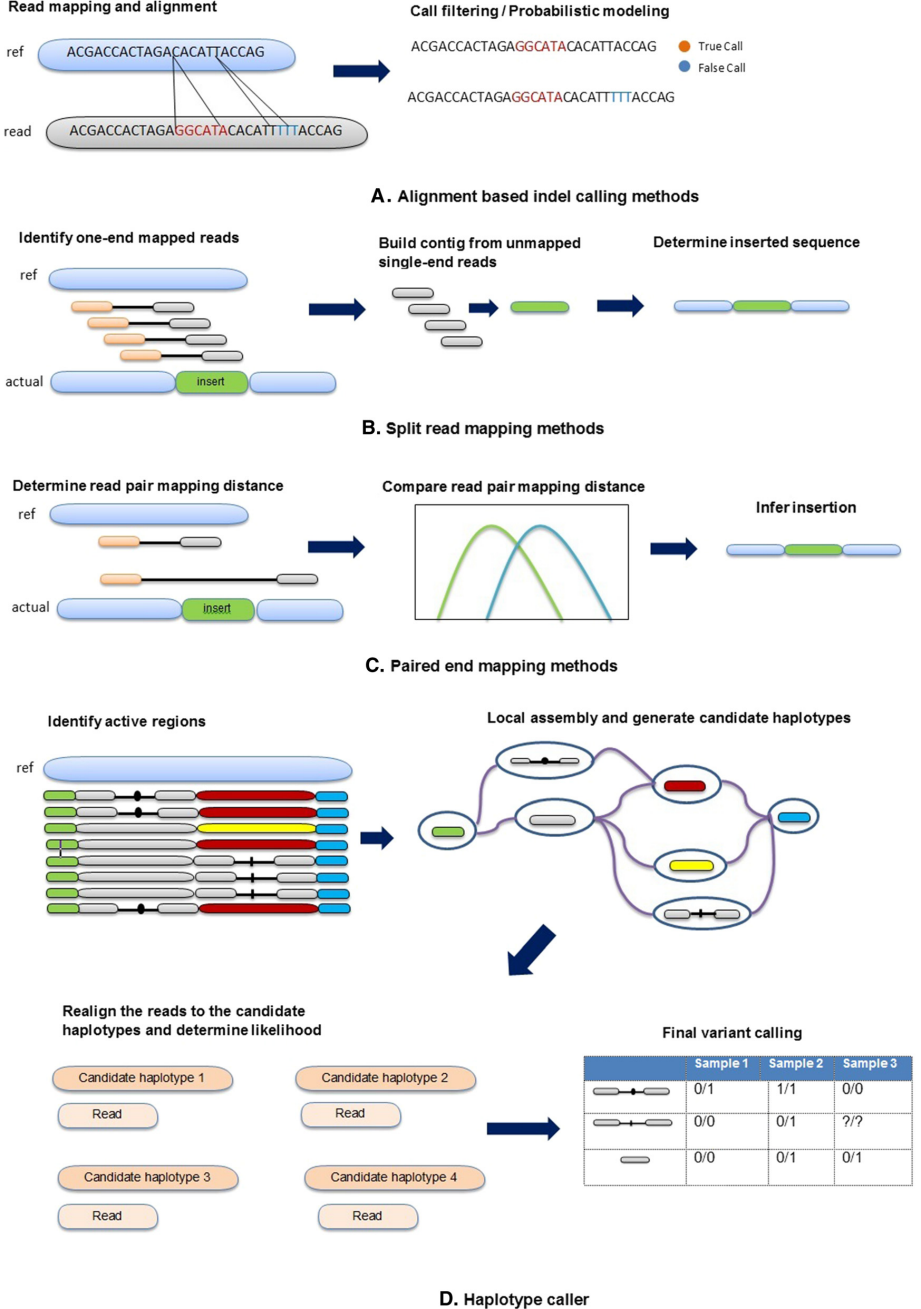
Categories of indel calling tools. Here *ref* is the reference sequence and *actual* is the observed sequence. (Partially taken from Abel and Duncavage [40])

A good number of indel calling tools have been developed so far that can be divided into four major categories: alignment-based methods, split read mapping methods, paired-end read mapping methods, and haplotype-based methods. Alignment-based methods firstly map the reads to the reference sequence using read mapping software such as BWA [24] and Novoalign [25], and then call indels using the alignment data by applying some filtering steps to separate true indels from common sequence alignment errors (Fig. 1 (A)). In Fig. 1 (A), “True Call” refers to the indels that passed after the filters are applied to separate indels from sequence alignment errors. Therefore, “False Calls” are those variants which are probably not indels but caused due to the alignment errors. Many indel calling tools belong to this category including Dindel [26], Stampy [27], SAMtools [28], Genome Analysis Tool Kit (GATK Unified Genotyper) [29], and VarScan [30, 31]. The main difference among these tools is in the model they use to distinguish true indel calls from alignment errors. Some use the Bayesian probabilistic model (GATK Unified Genotyper, SAMtools, and Dindel), whereas others (VarScan) use the heuristic approach. Split read mapping methods, on the other hand, firstly identify discordant paired-end reads for which one end maps completely to the reference sequence and the other end does not. The unmapped ends of these reads are then clustered or aligned by de novo assembly to determine indels (Fig. 1 (B)). Tools in this category include Pindel [32] that uses a pattern growth approach to detect breakpoints of indels, and SV-M [33] that performs a discriminative classification based on features of split read alignment profiles and then filters the result against empirically derived training set data to reduce the false-positive rate. Paired-end read mapping methods compare the expected distance to the actual mapped distance to determine whether there is any indel in the sequence (Fig. 1 (C)). Tools belonging to this category include PEMer [34], Hydra [35], and BreakDancer [36]. Haplotype-based methods first identify the regions of interest where the reads show substantial evidence of having indels relative to the reference sequence. These regions are also known as active regions. For each active region, the callers build a De Bruijn graph to reassemble the active regions and yield the possible haplotypes present in the reads. After that, each read is realigned to the possible haplotypes and the likelihood of the haplotypes are calculated given the read data. Later, Baye’s rule or EM algorithms are applied to calculate the posterior probabilities, and indels are called where the posterior probability exceeds a certain threshold value. In addition to that, some other filters are also applied to produce a fine-grained result. GATK HaplotypeCaller [37] and Platypus [38] belong to this category. Figure 1 (D) shows the general overview of the haplotype-based indel callers.

Despite many indel calling tools, evaluation of the tools objectively, particularly using large-scale real data, is sparse. There is an evaluation of four indel (Dindel, VarScan, GATK Unified Genotyper, and SAMtools) tools done by Neuman et al.; however, it was based on simulated data [39]. Instead of repeating the same experiment, here we performed the evaluation of the tools as well as three additional tools and we use real data to get the actual insight. In this study, we investigated seven indel calling tools, GATK Unified Genotyper [29], VarScan [30], Pindel [32], SAMtools [28], Dindel [26], GATK HaplotypeCaller [37], and Platypus [38], using 78 human genome data from different populations in the 1000 Genomes project. All these tools are publicly available and are commonly used for benchmarking. Another reason for choosing these tools is that GATK Unified Genotyper, VarScan, SAMtools, Dindel, GATK HaplotypeCaller, and Platypus can deal with short indels (<50 bps), whereas Pindel can call medium to large indels ranging from 50 to 10,000 bps. Therefore, altogether they cover indels of various lengths. Among these seven tools, four of them (GATK Unified Genotyper, VarScan, SAMtools, and Dindel) fall into the alignment-based method category, one (Pindel) implements the split read mapping method, and two (GATK HaplotypeCaller and Platypus) are haplotype-based methods. We did not consider tools that are based on paired-end read mapping because in most cases, they are insensitive to small indels, making it difficult to separate small perturbations in read pair distance from the normal background variability [40]. Moreover, the exact inserted or deleted sequence cannot be known from the results of tools that belong to this category [40]. We also note that only one of the two commonly used tools (Pindel and SV-M) from the split read mapping method category was included in this study. We did not consider SV-M mainly because this tool does not use BAM file as input. As described in the README file of SV-M, the input file requires the start and end position of each chromosome along with several features corresponding to that chromosome such as the number of uniquely mapped reads (UMRs) overlapping the deletion candidate, single position variation (SPV) from split read alignment, and number of split reads supporting the same indel location. For reason of consistency and to eliminate possible factors that could bias the comparison, we decide to exclude SV-M from this study.

## Methods

### Tools investigated

We investigated seven indel calling tools, GATK Unified Genotyper, VarScan, Pindel, SAMtools, Dindel, GATK HaplotypeCaller, and Platypus. A brief introduction of each tool and the commands for execution are provided below.

GATK Unified Genotyper (GATK_UG) [29] (version 2.7) is a tool developed by the Broad Institute of MIT and Harvard. For indel calling, it incorporates realistic read mapping error and base miscall models. Using a Bayesian genotype likelihood model, GATK_UG estimates the most likely genotypes and allele frequency in the sample while emitting an accurate posterior probability of having a segregating variant allele at each locus. We called indels by GATK_UG for each sample using the following command with default settings:



VarScan [30] (version 2.2.2) is a platform-independent software tool developed by the Genome Institute of Washington University. It uses the *mpileup* file generated by SAMtools [28] for scoring and sorting sequence alignments. The reads mapped uniquely to one location in the reference sequence are kept, whereas the unmapped and ambiguous mapped reads are discarded. The uniquely mapped reads are further filtrated on read depth, base quality, and variant allele frequency in downstream analysis and then used to call indels by a heuristic approach. Indels were called by VarScan with its default settings using the following commands:

Generating the mpileup file using SAMtools:

Calling indel from the mpileup file:
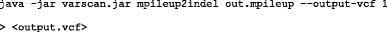


Pindel [32] (version 0.2.4) is a pattern growth approach-based tool that detects breakpoints of large deletions, medium-sized insertions, and other structural variants from NGS data at single-based resolution. In Pindel, all reads are initially mapped to the reference genome. The mapping results are then inspected to select paired reads that are mapped with indels or have only one end mapped. Based on the mapped reads, Pindel determines the anchor point on the reference genome as well as the direction of unmapped reads or the reads mapped with indels. Using this information and user-defined maximum deletion size, a sub-region in the reference genome is located where the unmapped reads are broken into fragments and then the fragments are mapped separately. Pindel was executed with its default settings using the following commands:

The configuration file:
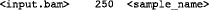


Here 250 is the insert size, i.e., the length of the region between the paired-end adapters in paired-end sequence.

Generating the output file:
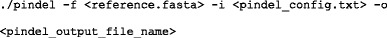


Creating the VCF file from the output file:
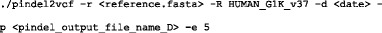


SAMtools [28] (version 0.1.19) is a software package used for parsing and manipulating alignments in SAM/BAM format. For indel calling, it uses a Bayesian model for local realignment and base quality assessment. We called indels by SAMtools with its default settings using the following command:

Generating the mpile file:
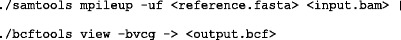


Calling indel from the mpileup file:
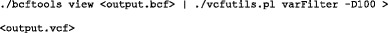


Dindel [26] (version 1.01), developed by the Wellcome Trust Sanger Institute in UK, is a software tool that uses Bayesian network for calling indels from NGS data. First, a number of candidate haplotypes, each containing at least 120 bps, are generated according to the hypothesis that indel events exist in pre-specified genomic segments. After realigning all reads to the candidate haplotypes using a hidden Markov model, the posterior probability of a haplotype is calculated using the Bayesian approach and used to determine the presence of indels in the sample. Dindel assumes that all differences between the read and the candidate haplotype are caused by sequencing errors. By realigning reads to the candidate haplotype, it separates the indels from sequencing errors. Dindel uses mapping quality as the prior probability that a read should align to any of the candidate haplotypes, and thus, it effectively reduces the weight of reads that cannot be confidently mapped to that location in the genome. We used Dindel with default settings to call indels by the following commands:

Step 1: Extract candidate indels from the alignment file.
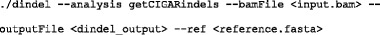


Step 2: Create realignment windows.
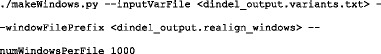


Step 3: For every window, generate candidate haplotypes from the candidate indels and realign the reads to these candidate haplotypes.

For each file created in step 2
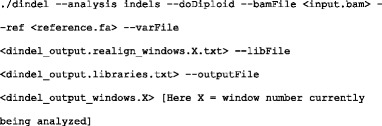


Step 4: Create the final output.

Merging results from all realignment windows:
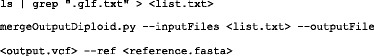


GATK HaplotypeCaller (GATK_HC) [37] (version 3.30) is a tool developed by the Broad Institute of MIT and Harvard. For indel calling, at first it determines the regions of the genome where there are significant evidences of variation. Therefore, regions that do not show any variation beyond the expected levels of background noise are skipped. After this step, the resulting regions having significant evidence of variations are passed to the next step. These regions are known as “Active Regions.” For each active region, in the second step, GATK_HC builds a De Bruijn graph to reassemble the active regions and identifies the candidate haplotypes present in the reads of the given sample. Additionally, each haplotype is locally realigned to the reference haplotype to identify the potentially variant sites. In the next step, for each active region, each read is then pairwise aligned to each of the candidate haplotype using the PairHMM algorithm. This produces a matrix of likelihoods of haplotypes for the reads in the given sample. These likelihoods are then marginalized to obtain the likelihoods of the alleles per read for each potentially variant site. For each potentially variant site, in the next step, Baye’s rule is applied to determine the posterior likelihoods of each genotype per sample using the likelihoods of alleles obtained in the previous step. The most likely genotype is then assigned to the given sample. We called indels using GATK_HC with default settings using the following command:
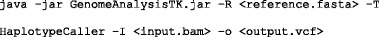


Platypus [38] (version 0.7.9.1) is a haplotype-based variant calling tool developed by the Wellcome Trust Sanger Institute in UK. In this tool, at the beginning, candidate variants are obtained from read alignments, local assembly, and external sources, and then candidate haplotypes are formed. After haplotypes are generated from candidate variants, their frequencies are estimated on the basis of their likelihood. These likelihoods are calculated by aligning a read to the haplotype sequence with an underlying hidden Markov model (HMM). The forward algorithm is used to calculate the likelihood of a read given haplotype. After the likelihood is calculated for all combinations of reads and haplotypes, an EM algorithm is used to estimate the frequency of each haplotype under a diploid genotype model. In the next step, the posterior support for any variant is computed by comparing the likelihood of the data given all haplotypes and the likelihood given only those haplotypes that do not include a particular variant. Later, indels are called when their posterior support exceeds a threshold using these frequencies as a prior. The variants are also filtered based on allele bias, strand bias, mapping quality, quality over depth, posterior quality, and sequence context. We called indel with the default settings of Platypus using the following command:
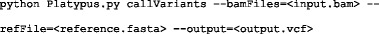


### Dataset

The dataset consists of low-coverage (~3X to ~12X) alignment profiles from 78 humans that belong to 26 populations and were collected for the 1000 Genomes project [41]. We used the alignment files of chromosome 11 as input for the tools we investigated. These short reads were sequenced on Illumina Genome Analyzer platform [42] and mapped using BWA [24]. We used hs37d5 as the human reference genome, which is an extended version of the Build37 dataset of the 1000 Genomes project with additional sequences. Note that this reference genome was used by the 1000 Genomes project in the final phase. Additional file 1: Table S1 lists the samples we used with their corresponding ethnic background and coverage.

Ideally, a benchmarking dataset for evaluating indel calling tools would consist of a list of known indels for the samples. However, such kind of benchmarking dataset is not available in large quantity [43]. Hence, for evaluation purpose, we used the indels identified in Mills et al. [43] as the gold standard. To call indels, Mills et al. [43] examined 98 million Applied Biosystems (Sanger) DNA re-sequencing traces from the trace archive of NCBI which has been proved to be sufficient for accurate indel calling [4]. After some pre-processing of the traces based on the quality scores, they were compared to the human reference genome to call indels. Details about the indel calling procedure and some post processing to generate the gold standard dataset can be found in [4]. The called indels were validated using PCR-based methodologies, and the validation rate was 97.2 %. This dataset reports almost two million small and large indels found in all 24 chromosomes of 79 diverse humans with length ranging from 1 to 10,000 bps. Moreover, it has been confirmed that the sequence traces used in Mills et al. provide excellent coverage of the human genome [43]. Note that the samples we used here are sequenced on Illumina Genome Analyzer platform and the indels listed in the “gold standard” dataset are called using the Applied Biosystem (Sanger) DNA re-sequencing traces. In spite of these differences, the indels identified in the gold standard dataset are considered to be most likely reliable, and they have been used as the gold standard in other studies [44, 45]. In Mills et al. [43], 58,811 indels were identified for chromosome 11, and in the current study, we used this set as the gold standard. Note that we did not use simulated data for benchmarking because though simulated data are valuable, they do not always represent the actual phenomena. We could also use the sample benchmark dataset available in “Genome in a Bottle Consortium” [46], but that one relies on a single dataset from one human only (NA12878).

### Evaluation criteria

We evaluated the tools using the criteria including running time, number of indels called, comparison with the set of gold standard indels, similarity among the tools, hierarchical clustering, and ranking of the tools.

For each sample, we executed the tools and recorded the number of indels called by each tool as well as the running time. To see the relation between running time and coverage of the read, besides the low-coverage samples, we also included the sample NA12878 with ~64X coverage. All analyses were done on a Linux machine with Intel Core i7-2600 CPU @ 3.40 GHz * 8 processors, 16 GB RAM and Ubuntu 12.04 LTS operating system.

Indels called by the seven tools were compared with those identified in Mills et al. [43]. From this comparison, we calculated the corresponding recall and precision for each of the tools using formulas (1) and (2).

1 $$ \mathrm{R}\mathrm{e}\mathrm{call}\kern0.5em =\kern0.5em \frac{\mathrm{TP}}{\mathrm{TP}+\mathrm{F}\mathrm{N}} $$

2 $$ \Pr \mathrm{ecision}=\frac{\mathrm{TP}}{\mathrm{TP}+\mathrm{F}\mathrm{P}} $$

For comparing the accuracy of the tools, we used F-measure, the harmonic mean of the precision and recall, where an F-measure reaches its best value at 1 and worst score at 0. The F-measure was calculated using formula (3).

3 $$ \mathrm{F}\hbox{-} \mathrm{measure}=\frac{2\kern0.5em \times \kern0.5em \mathrm{Recall}\times \kern0.5em \mathrm{Precision}}{\mathrm{Recall}+\mathrm{Precision}} $$

**Fig. 2:**
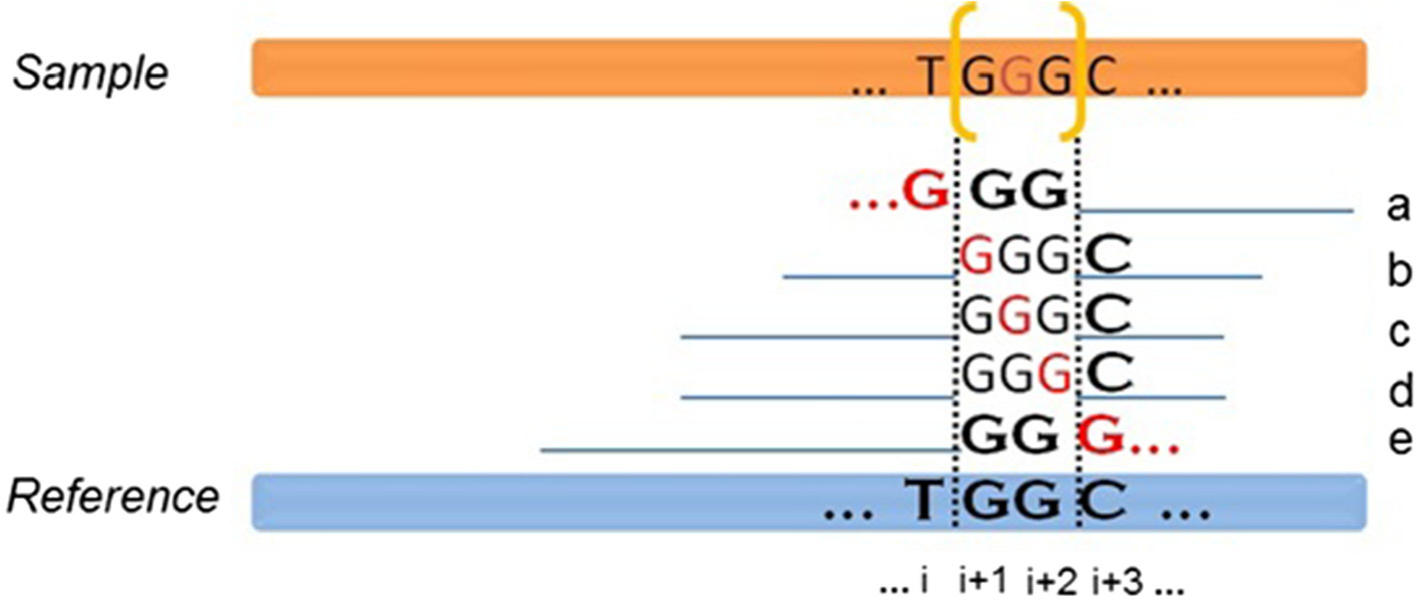
Example of identical indels taking place in relative positions. (Adapted from Krawitz et al. [20])

Note that the position of an indel with respect to the reference sequence sometimes cannot be defined unambiguously by a single coordinate [20, 47]. As shown in Fig. 2, the insertion of a guanine into the local sequence of *T*_*i*_*G*_*i* + 1_*G*_*i* + 2_*C*_*i* + 3_ after position *i* produces the same mutated sequence as inserting guanine after position *i* + 1 or *i* + 2. Hence, these insertions have identical biological meaning, and therefore, an unambiguous annotation for this insertion should list all equivalent indel positions, i.e., +*G* {*i*, *i* + 1, *i* + 2} [20]. For this reason, while comparing an indel called by each tool with the indel in position *i* in the gold standard data, we treated the indel called by the tool as true positive if it is within the range of *i* ± 5 positions.

Based on the indel calling results, these tools were ranked in the receiver operating characteristic (ROC) space [48], where the *X* and *Y* axes are denoted by false-positive rate (FPR) and true-positive rate (TPR), respectively. Here TPR is equivalent to recall and FPR is simply (1 − precision) as calculated using formulas (1) and (2). In the ROC space, each point represents the prediction result or instance of a confusion matrix. The diagonal (*Y* = *X*) that divides the ROC space represents the decision from a “Random Guess.” Points above the diagonal represent good classification results, whereas points below the line represent poor results. For each sample, we first calculated the TPR and FPR for each tool and plotted as a point in the ROC space, then ranked the tools based on the perpendicular distance of each point from the diagonal.

We also examined the similarity among the results produced by different tools. Jaccard index, also known as Jaccard similarity coefficient, is used to compare the similarity between indel predictions. For two finite sets *A* and *B*, the Jaccard index can be calculated using

4 $$ J\left(A,\kern0.5em B\right)=\kern0.5em \frac{\left|A\cap B\right|}{\left|A\cup B\right|},0\le \kern0.5em J\left(A,\kern0.5em B\right)\le \kern0.5em 1. $$

The maximum value of the Jaccard index is 1 when two indel sets are the same, whereas the minimum is 0 when two indel sets are completely different.

Another interesting question to ask is “how are the seven indel calling tools related to one another on the whole”? To answer this question, we clustered the tools using the following three steps: (1) Divide the reference sequence into windows of equal size. We tested with different window sizes (1000, 10,000, 100,000, and 1,000,000 bps) and found that the window size does not affect the clustering result. For computational convenience, we set the window size to 1,000,000 bps. (2) For each window, calculate the number of indels called by each tool. (3) Construct a vector of indel counts of all windows for each tool and apply the UPGMA hierarchical clustering algorithm to the seven vectors.

## Results

### Running time

We compared the tools on the average running time taken to call indels for a sample. Table 1 shows the average running time for samples with low coverage (average coverage ~6X) and high coverage (~64X). For both high- and low-coverage data, Platypus is the fastest and Dindel is the slowest of all the tools investigated. Clearly, indel calling is more time consuming for high-coverage data than for low-coverage data, which is especially evident for Dindel due to its complicated model for realignment. Since Dindel tests all indels identified by the read mapper, many of which might be sequencing errors, with the increase of number of reads and increase in sequencing errors, the computation time increases quadratically [26].

**Table 1:** Average running time spent in calling indels for samples with low/high coverage

Tool	Time	
	Low Coverage (~6X)	High Coverage (~64X)
GATK_UG	16 minutes 43 seconds	24 minutes 19 seconds
VarScan	16 minutes 1 second	84 minutes 02 seconds
Pindel	25 minutes 36 seconds	139 minutes 09 seconds
SAMtools	11 minutes 26 seconds	64 minutes 14 seconds
Dindel	165 minutes 22 seconds	1549 minutes 18 seconds
GATK_HC	58 minutes 27 seconds	91 minutes 13 seconds
Platypus	3 minutes 36 seconds	5 minutes 59 seconds

### Number of indels called

**Fig. 3:**
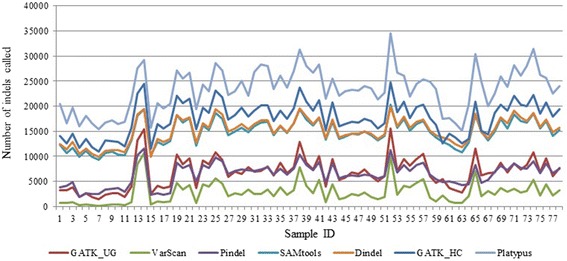
Number of indels called by the seven tools for the 78 humans

Figure 3 shows the number of indels called by each tool for each sample. The seven tools under consideration call different numbers of indels. The numbers of indels called across the 78 samples range from 1431 to 15,585 for GATK_UG, from 114 to 10,619 for VarScan, from 1845 to 11,455 for Pindel, from 9351 to 20,245 for SAMtools, from 9864 to 19,876 for Dindel, from 10,915 to 24,786 for GATK_HC, and from 15,062 to 34,600 for Platypus. On average, Platypus calls the maximum number of indels (average number = 23,321), whereas VarScan calls the minimum (2,775). The average number of indels called by SAMtools (14,719) follows closely to that by Dindel across the samples. Similarly, the average numbers of indels called by GATK_UG (6733) and Pindel (6382) are very similar to each other across the samples. As we can see from these results, VarScan is evidently the most conservative one in calling indels. It calls much fewer indels than others. This might be due to its rather stringent filtering step during which all the unmapped and ambiguous reads are discarded. Although this step is helpful in keeping the false positives down, it also reduces the power of detecting true indels.

### The lengths of indels called

We examined the distributions of lengths of indels called by the seven tools and compared them to that of the gold standard dataset. All the indel distributions based on lengths are shown in Additional file 1: Figure S1 which shows that 96.2 % of the indels in the benchmark dataset are 1–10 bps, 98.6 % in GATK_UG, 99.0 % in VarScan, 93.4 % in Pindel, 92.2 % in SAMtools, 95.1 % in Dindel, 94.01 % in GATK_HC, and 97.19 % in Platypus. Therefore, most of the indels in the benchmark and the ones called by the tools are ≤10 bps. Chi-square statistical tests show that the distributions of indel sizes are not significantly different between the calling results of the tools and the gold standard (*p* values for comparing the gold standard with GATK_UG, VarScan, Pindel, SAMtools, Dindel, GATK_HC, and Platypus are 0.89, 0.81, 0.96, 0.28, 0.94, 0.95, and 0.99, respectively). Note that Pindel is known for calling medium to large indels, but here most of the indels called by Pindel are small indels.

Regardless of the gold standard indels, we are interested to see the similarity/dissimilarity of the distribution of indel sizes among the tools themselves. From chi-square statistical test between intra-tools, we see that the distributions of indel sizes are not significantly different among the tools. The *p* values for intra-tool comparisons are showed in Additional file 1: Table S2.

### Effect of the depth of coverage on the number of indels called

**Fig. 4:**
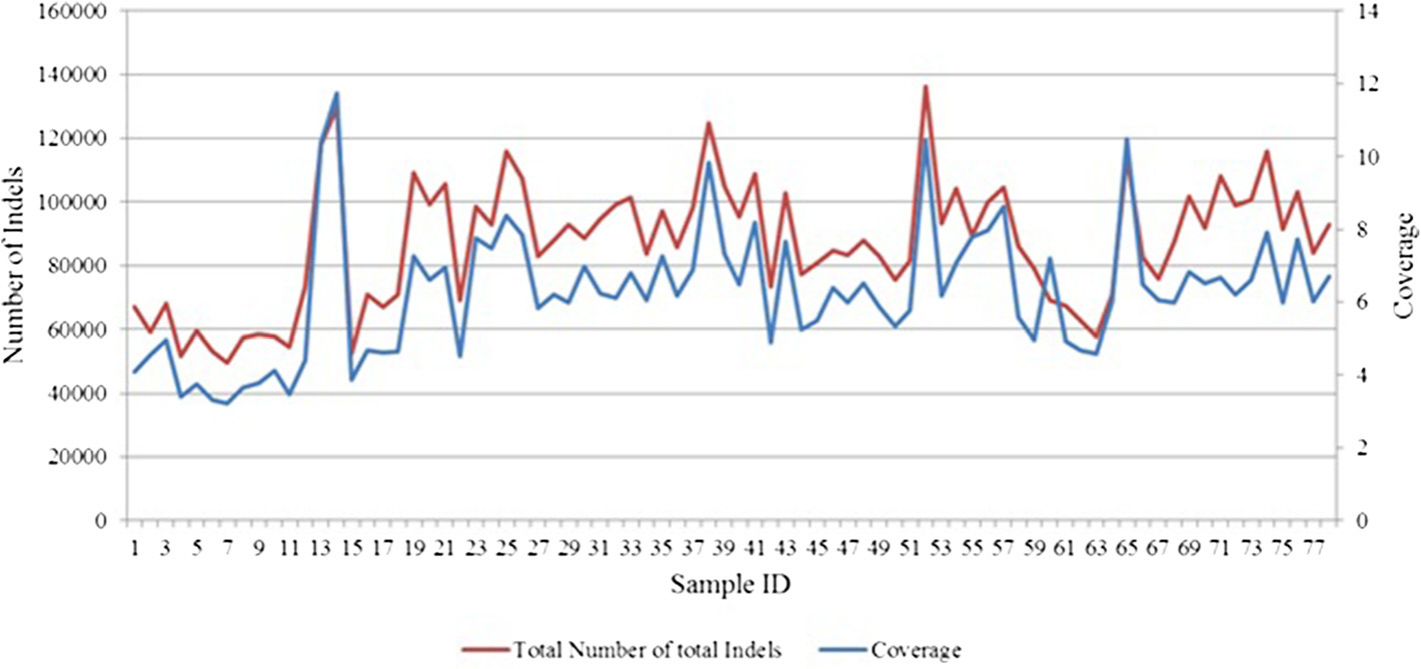
Relationship between coverage and the pooled number of indels by the seven tools

To see how the number of indels called by these tools is affected by the depth of coverage, we estimated the depth of coverage for each human sample (shown in Additional file 1: Table S1). Figure 4 shows the relationship between the number of indels called by the seven tools and the coverage depth. Overall, the higher the coverage is, the more indels are called. Pearson correlation coefficients between the coverage and the number of indels called by GATK_UG, VarScan, Pindel, SAMtools, Dindel, GATK_HC, and Platypus are 0.97 (*p* value = 8.02 × 10^−48^), 0.97 (*p* value = 5.25 × 10^−48^), 0.91 (*p* value = 3.10 × 10^−30^), 0.89 (*p* value = 4.64 × 10^−27^), 0.88 (*p* value = 2.76 × 10^−26^), 0.86 (*p* value = 2.24 × 10^−23^), and 0.82 (*p* value = 6.84 × 10^−20^), respectively. Thus, consistent with previous findings [49], the number of indels called, regardless of the tools, is significantly positively correlated with the coverage depth.

### Comparison with the set of “gold standard” indels and ranking of the tools

**Fig. 5:**
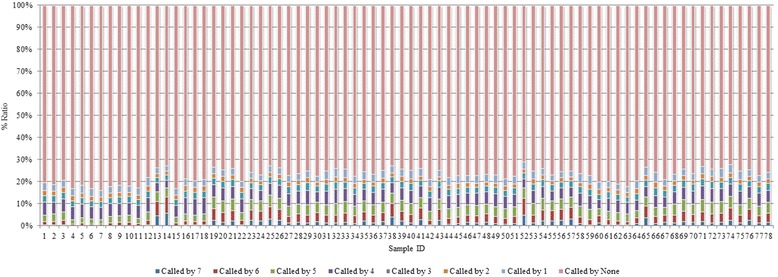
Percentage of the gold standard indels called by the tools

Figure 5 shows the percentage of gold standard indels called by the tools across the 78 samples. For chromosome 11, on average, only about 1.51 % of the gold standard indels are called by all seven tools, whereas about 76.91 % are undetected by any of the tools. The remaining ~21.58 % are called by at least one tool. We also compared the tools for the percentage of their own indels called by others regardless of the gold standard indels. For this purpose, we picked up Dindel, SAMtools, GATK_HC, and Platypus as they call more indels than the other three tools. The Venn diagram in Additional file 1: Figure S2 shows that only 15.64 % of the indels were called by all of these four tools revealing that regardless of the gold standard indels, a major percentage of indels remain undetected.

We also examined the overall performance of each tool on the 78 samples. The average F-measure values for GATK_UG, VarScan, Pindel, SAMtools, Dindel, GATK_HC, and Platypus are 0.14, 0.06, 0.12, 0.26, 0.27, 0.28, and 0.31, respectively.

In the ROC analysis, we ranked the seven tools based on their distance from the “Random Guess” line in the ROC space. Table 2 shows the frequency of the ranks of the tools based on the 78 samples. Platypus ranked the best for all 78 samples, and GATK_HC ranked the second best. VarScan performed poorly, ranking the worst for 76 samples. Pindel performed also poorly, ranking the worst for 2 samples and second worst for 60 samples. In addition to the ranking, we also computed the average recall, precision, and F-measure for the tools in Table 2. For the average recall of the 78 samples, Platypus ranks the highest (0.22), followed closely by GATK_HC (0.18), and VarScan the lowest (0.03). For the average precision, GATK_UG ranks the highest (0.72), followed closely by VarScan (0.71). GATK_HC (0.61) and Platypus (0.56) have slightly lower average precision. For the average F-measure, Platypus (0.31) ranks the highest and VarScan (0.06) the lowest. To get a clear idea about how the performance of the tools depends on the indel types, i.e., insertion and deletion, we split the benchmark dataset based on the indel types and results are shown in Additional file 1: Figure S3. Results show that except Pindel, performance of the other tools remains consistent regardless of the indel type. Pindel shows better performance in calling deletion than insertion.

**Table 2:** Frequency of the ranks of the tools based on the ROC curve for the 78 samples. Average recall, precision, and F-measure across the samples are also provided

Rank	1	2	3	4	5	6	7	Average Recall	Average	Average
Name									Precision	F-measure
GATK_UG	0	0	0	0	62	16	0	0.081884	0.72141	0.14435
VarScan	0	0	0	0	0	2	76	0.033987	0.717315	0.063333
Pindel	0	0	0	0	16	60	2	0.068635	0.636704	0.122591
SAMtools	0	0	1	77	0	0	0	0.160989	0.645108	0.256343
Dindel	0	5	73	0	0	0	0	0.170076	0.662287	0.269404
GATK_HC	0	73	4	1	0	0	0	0.181928	0.608907	0.278323
Platypus	78	0	0	0	0	0	0	0.220391	0.559842	0.314071

### Performance of the tools on indels of different lengths

**Fig. 6:**
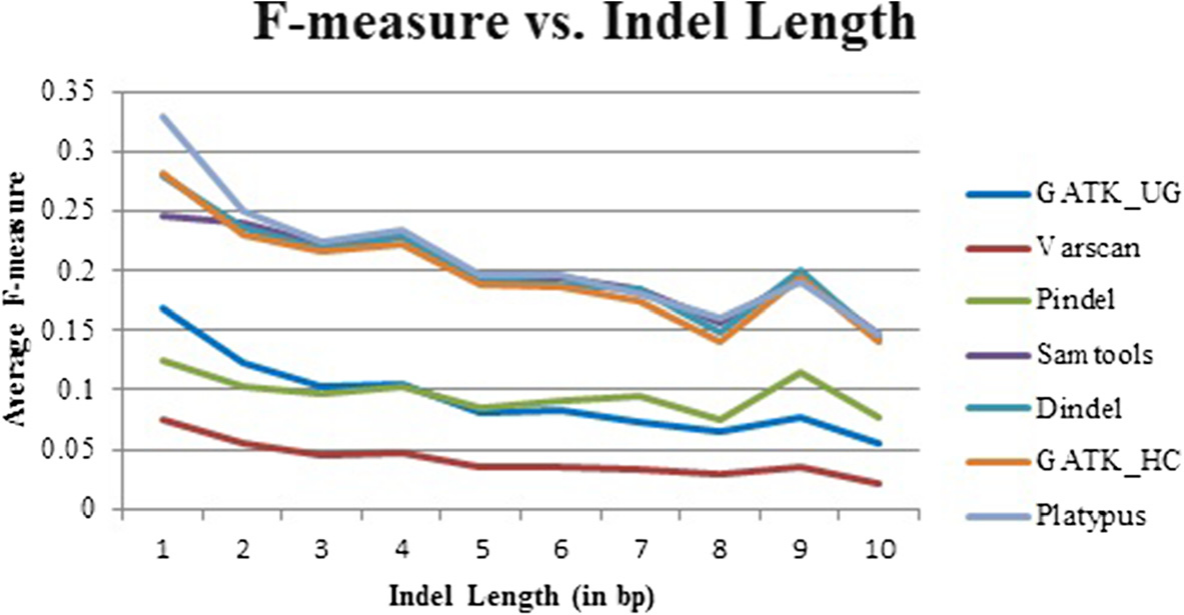
Average F-measure for each tool for different lengths of indels

**Fig. 7:**
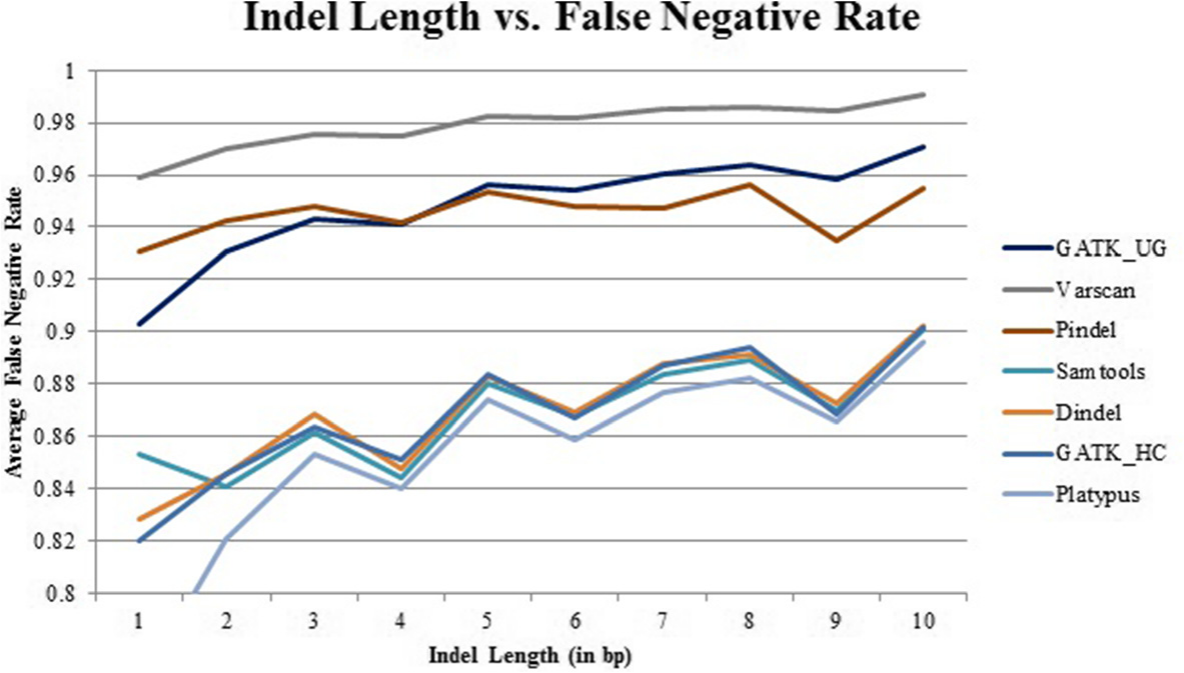
Average false-negative rate for each tool for different lengths of indels

A natural question to ask is whether the seven tools’ performance changes with different indel sizes. We computed the average F-measure (Fig. 6), false-negative rate (Fig. 7), recall (Additional file 1: Figure S4), and precision (Additional file 1: Figure S5) of the seven tools for indels of lengths 1–10 bps. Results show that for all the tools, the performance of calling indels correctly shows a slight decrease with the increase of indel lengths. Platypus, GATK_HC, Dindel, and SAMtools show highly similar patterns for four metrics (i.e., F-measure, false-negative rate, recall, and precision) with respect to indel lengths. Altogether, this comparison based on indels of different lengths shows that these tools achieve similar performance for different subcategories of indels with certain length. In other words, indel length is not a confounding factor that affects the performance of these calling tools.

### Similarity among the tools

**Fig. 8:**
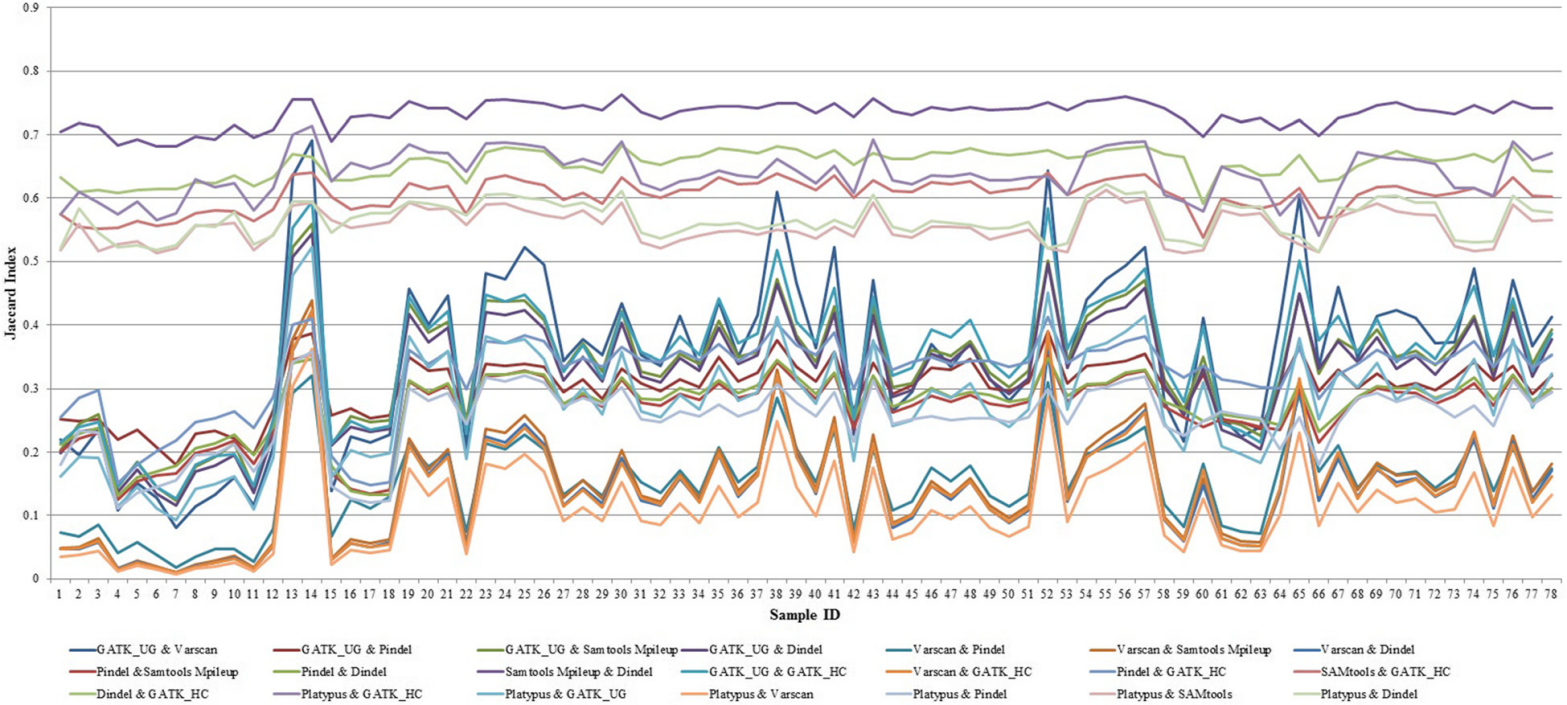
Jaccard index for each pair of tools for each sample

We also compared the tools for their similarity regardless of the gold standard. For each sample, the Jaccard index of each pair of the tools is shown in Fig. 8, and the average Jaccard index across all samples is listed in Table 3. From the Jaccard index, we found high similarity between SAMtools and Dindel. A possible reason is that both tools use the Bayesian approach for calling indels. SAMtools calculates the Bayesian prior probability and uses it to calculate the actual genotype for the variants detected. Dindel, on the other hand, calls indels by realigning the reads against candidate haplotypes for which prior probabilities calculated using the Bayesian approach are already known. Both SAMtools and Dindel perform local realignment and base quality assessment for calling indels, and that is also another possible reason for their similarity. Similarly, Platypus and GATK_HC also have high Jaccard index value that represents their strong similarity. Being a haplotype caller, they have underlying similarity such as generating candidate haplotypes and then realigning reads to each of these candidate haplotypes for variant calling which explains the reason of their similarity.

**Table 3:** Average Jaccard index for each pair of the tools (Jaccard index is computed for each pair of the tools for each human sample and then averaged across all the 78 samples)

	GATK_UG	VarScan	Pindel	SAMtools	Dindel	GATK_HC	Platypus
GATK_UG	1	0.35	0.30	0.34	0.32	0.35	0.28
VarScan		1	0.15	0.15	0.14	0.14	0.11
Pindel			1	0.27	0.27	0.32	0.25
SAMtools				1	0.73	0.60	0.56
Dindel					1	0.65	0.57
GATK_HC						1	0.64
Platypus							1

**Fig. 9:**
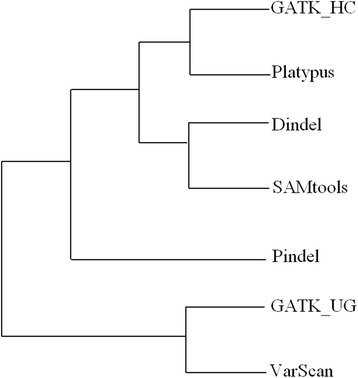
Hierarchical clustering of the tools

Figure 9 shows the dendrogram on hierarchical clustering of the tools. Again we see that Dindel and SAMtools group together and Platypus and GATK_HC group together which is supporting our previous observation of similarity between these tools.

## Discussion

In this paper, we investigated seven tools that are publicly available and well known for calling indels from short reads. Using 78 whole genome short-read data from the 1000 Genomes project, we evaluated these tools based on several criteria, including running time, number of indels called, recall, precision, F-measure based on the “gold standard” data, and ranking and clustering of the tools. Results show that Platypus outperforms other tools in most of the aspects.

The low percentage of the called indels over the “gold standard” indels indicates that all these tools exhibit limited power in detecting indels. Several factors could contribute to the low true-positive rate. Firstly, since existing read mappers map each read to the reference sequence independently of other reads, due to the alignment artifacts, insertions and deletions can be improperly placed relative to their true positions and it affects the indel calling results greatly. Secondly, most of the indel calling tools do not have sophisticated methods for checking sequencing errors before calling indels. Though Platypus, GATK_HC, and Dindel realign the candidate indels to the known haplotypes, especially for Dindel and GATK_HC, due to their high computational time, it is not an efficient way when the depth of coverage of the reads is high. Therefore, indel calling results can be improved if these factors are considered. Thirdly, the indels we used as gold standard were identified from the DNA traces obtained from the trace archive at NCBI [50], and though it is more reliable than using short reads, indels identified in this way nevertheless can still be false positives, which could lead to an artificial decrease of true-positive rate. Fourthly, the set of gold standard indels is the pooled result of indels from 79 individuals, which naturally has more indels than individual humans. However, this might not be the dominating factor causing the low false-positive rate as the number of pooled indels for 78 humans is still very low compared to the “gold standard”. Finally, the low true-positive rate might also be due to the chromosome-specific behavior of the calling tools. Although we have no particular reason to suspect that the indel calling results for chromosome 11 should be different from those for other autosomes, we examined the performance of the seven tools on chromosome 20 to see whether the result is chromosome specific. Results show that all the metrics (i.e., recall, precision, and F-measures) follow closely those of chromosome 11 (Additional file 1: Figure S6), and therefore, the poor performance of the tools evaluated by the gold standard indels is not chromosome specific.

Clearly, an important issue in evaluating various indel calling tools is the lack of a gold standard dataset or benchmark dataset. In the current study, the performance comparison is done based on the “gold standard” dataset that is the best possible resource available. Although it lists two million short and long indels extracted from the genomes of 79 diverse human, it does not list all the indels that take place in the genomes of the human samples we considered here. Though we can say that Platypus performs better than other tools based on the “gold standard” dataset, however, in general, we cannot make a decision about which tool is the best unless we have the list of true indels for each sample. So developing a list of indels for individual humans will be a good direction for future research, and that list will be a useful resource for validating the existing as well as newly developed indel calling tools. Moreover, people from the same ethnic group tend to have common indels [51, 52]. Therefore, creating a list of known indels for the same ethnic group and comparing the tools based on the indels called for the samples from that ethnic group would be a better way to evaluate the performance of the tools.

Besides improving the indel calling tools, another strategy to improve the indel calling result is increasing the depth of coverage of the reads. For each of the tools, the performance shows positive correlation with the coverage of the reads. Pearson correlation coefficients between coverage and F-measure for GATK_UG, VarScan, Pindel, SAMtools, Dindel, GATK_HC, and Platypus are 0.96 (*p* value = 6.75 × 10^−44^), 0.98 (*p* value = 1.38 × 10^−54^), 0.89 (*p* value = 1.56 × 10^−27^), 0.87 (*p* value = 1.22 × 10^−24^), 0.85 (*p* value = 3.65 × 10^−23^), 0.85 (*p* value = 9.47 × 10^−23^), and 0.81 (*p* value = 1.15 × 10^−19^), respectively. Moreover, we also performed down-sampling of the individual that has 64X coverage to create a 5X coverage sample and conducted indel calling using the seven tools. Results further confirm that higher coverage yields better results, reflected by higher F-measures for all seven tools in the 64X coverage. However, for all seven tools, precision is higher in the 5X coverage sample than in the 64X coverage sample. Detailed results are shown in Additional file 1: Figure S7. Hence, the performance of the tools can be significantly improved by increasing the depth of coverage of the reads. Consistent with our finding, a previous evaluation of indel calling tools based on simulation data has shown that the sensitivity of indel calling tools increases with coverage depth [39]. Joint sample calling is another strategy to call indels from low-coverage data, and greater sensitivity can be achieved through this. However, it has a few limitations as follows: (i) Since it calls variants simultaneously across all samples, computational expense increases exponentially with the increase of the number of samples, and (ii) every time a new sample is added to the cohort, the process of variant calling needs to start again from the scratch; this is known as the (*N* + 1) problem [53]. HaplotypeCaller like GATK_HC and Platypus are free from these limitations; however, the other tools are yet to overcome these limitations.

Finally, although the indel calling results produced by the tools show great discrepancy, these tools can show strengths in different aspects such as running time, the number of indels identified, and indels of different lengths. Thus, integrating the strength of existing tools to call indels and then passing the results to an aggregating machine learning model to increase true positives and reduce false positives might be a good solution. Similar ideas were discussed in [54] for creating highly confident SNP, indel, and homozygous reference genotype calls.

## Conclusion

Indel is one of the main types of disease-causing variation in humans. Detecting indels in an efficient manner is necessary for discovering proper medication. The advent of NGS technology has made it possible to sequence human genomes at an individual level. We have investigated seven well-known tools, GATK Unified Genotyper (GATK_UG), VarScan, Pindel, SAMtools, Dindel, GATK HaplotypeCaller (GATK_HC), and Platypus that call indels using NGS data. Based on the benchmark dataset we used, Platypus outperformed other tools. However, all of these tools have limitations as a large number of indels listed in the benchmark dataset remain undetected. A sophisticated method to check sequencing errors before calling indels and an integrative approach to combine the strengths of existing indel calling tools might be a good solution to overcome this problem. Using reads with high coverage is another strategy to obtain better results. Although the benchmark dataset we used for comparing the tools contain a large number of short and long indels that take place in diverse human genomes, it may not contain all the indels occurring in the genome of the samples we considered here. Hence, developing a list of known indels at an individual level will be helpful for validating the existing and newly developed tools.

## Additional file

Additional file 1: **Supplementary materials for Performance evaluation of indel calling tools using real short-read data.** This additional file includes List of the input samples used in this study with corresponding population and read coverage, P-value for Chi-square statistical test of indel size distribution between the tools, Distribution of indels based on lengths (1 to 10 bp) for the tools, Intra-tool comparison among GATK_HC, Dindel, SAMtools, and Platypus for percentage of their own indels called by others, Average Recall, Precision, and F-Measure of each tool for insertion and deletion, average recall and precision for each tool for different lengths of indels, Comparison between Chromosome 11 and Chromosome 20 for HG00157, and Comparison between High (~64X) and Low (~5X) coverage samples for NA12878.

## Abbreviations

HC: HaplotypeCaller
Indel: insertion and deletion
NGS: next-generation sequencing
ROC: receiver operating characteristic
SNP: single nucleotide polymorphism
WGS: whole genome sequencing
