# Supplementary material for: Performance evaluation of indel calling tools using real short-read data
Source: Hum Genomics. 2015 Aug 19;9(1):20. doi: 10.1186/s40246-015-0042-2 (PMC4545535; doi:10.1186/s40246-015-0042-2)
Supplement: Supplementary file 1 — Supplementary materials for Performance evaluation of indel calling tools using real short-read data. This additional file includes List of the input samples used in this study with corresponding population and read coverage, P-value for Chi-square statistical test of indel size distribution between the tools, Distribution of indels based on lengths (1 to 10 bp) for the tools, Intra-tool comparison among GATK_HC, Dindel, SAMtools, and Platypus for percentage of their own indels called by others, Average Recall, Precision, and F-Measure of each tool for insertion and deletion, average recall and precision for each tool for different lengths of indels, Comparison between Chromosome 11 and Chromosome 20 for HG00157, and Comparison between High (~64X) and Low (~5X) coverage samples for NA12878. [file 40246_2015_42_MOESM1_ESM.pdf]

Supplementary Material

Performance evaluation of indel calling tools using real short-read data

Mohammad Shabbir Hasan[1], Xiaowei Wu[2], Liqing Zhang[1*]

[1]Department of Computer Science

[2]Department of Statistics

Virginia Tech., Blacksburg, VA 24061

E-mail: {shabbir5, xwwu, lqzhang}@vt.edu

* - Corresponding Author

Supplemental Tables:

Supplemental Table S1: List of the input samples with corresponding population and read coverage

Sample ID	Sample	Ethnic Background	Average Coverage
1	HG00151	GBR (British in England and Scotland)	4.07945
2	HG00154	GBR (British in England and Scotland)	4.55756
3	HG00157	GBR (British in England and Scotland)	4.97155
4	HG00171	FIN (Finnish in Finland)	3.42147
5	HG00174	FIN (Finnish in Finland)	3.75145
6	HG00190	FIN (Finnish in Finland)	3.3264
7	HG00553	PUR (Puerto Rican in Puerto Rico)	3.20752
8	HG00560	CHS (Han Chinese South)	3.6613
9	HG00565	CHS (Han Chinese South)	3.77876
10	HG00580	CHS (Han Chinese South)	4.11136
11	HG00637	PUR (Puerto Rican in Puerto Rico)	3.46052
12	HG00638	PUR (Puerto Rican in Puerto Rico)	4.41764
13	HG00844	CDX (Chinese Dai in Xishuangbanna, China)	10.4013
14	HG00851	CDX (Chinese Dai in Xishuangbanna, China)	11.7362
15	HG00864	CDX (Chinese Dai in Xishuangbanna, China)	3.86342
16	HG01356	CLM (Colombian in Medellin, Colombia)	4.68011
17	HG01357	CLM (Colombian in Medellin, Colombia)	4.63414
18	HG01377	CLM (Colombian in Medellin, Colombia)	4.65449
19	HG01524	IBS (Spanish Iberian populations in Spain)	7.26326
20	HG01525	IBS (Spanish Iberian populations in Spain)	6.60486
21	HG01531	IBS (Spanish Iberian populations in Spain)	6.93617

22	HG01599	KHV (Kinh in Ho Chi Minh City, Vietnam)	4.52677
23	HG01860	KHV (Kinh in Ho Chi Minh City, Vietnam)	7.76182
24	HG01861	KHV (Kinh in Ho Chi Minh City, Vietnam)	7.47835
25	HG01882	ACB (African Caribbean in Barbados)	8.37547
26	HG01883	ACB (African Caribbean in Barbados)	7.84466
27	HG01892	PEL (Peruvian in Lima, Peru)	5.82321
28	HG01893	PEL (Peruvian in Lima, Peru)	6.20132
29	HG01914	ACB (African Caribbean in Barbados)	5.9745
30	HG01923	PEL (Peruvian in Lima, Peru)	6.97538
31	HG02813	GWD (Gambian in Western Division, The Gambia)	6.23018
32	HG02814	GWD (Gambian in Western Division, The Gambia)	6.10095
33	HG02839	GWD (Gambian in Western Division, The Gambia)	6.80767
34	HG03009	BEB (Bengali in Bangladesh)	6.04669
35	HG03234	PJL (Punjabi in Lahore, Pakistan)	7.25371
36	HG03235	PJL (Punjabi in Lahore, Pakistan)	6.17131
37	HG03268	ESN (Esan in Nigeria)	6.89912
38	HG03470	MSL (Mende in Sierra Leon)	9.82357
39	HG03473	MSL (Mende in Sierra Leon)	7.319
40	HG03514	ESN (Esan in Nigeria)	6.48069
41	HG03515	ESN (Esan in Nigeria)	8.20044
42	HG03571	MSL (Mende in Sierra Leon)	4.89722
43	HG03619	PJL (Punjabi in Lahore, Pakistan)	7.67182
44	HG03645	STU (Sri Lankan Tamil in the UK)	5.22783
45	HG03646	STU (Sri Lankan Tamil in the UK)	5.4842
46	HG03777	ITU (Indian Telugu in the UK)	6.40374
47	HG03785	ITU (Indian Telugu in the UK)	5.9841
48	HG03790	ITU (Indian Telugu in the UK)	6.51514
49	HG03808	BEB (Bengali in Bangladesh)	5.84893
50	HG03809	BEB (Bengali in Bangladesh)	5.32334
51	HG03999	STU (Sri Lankan Tamil in the UK)	5.76619
52	NA07000	CEU (Utah residents with Northern and Western European ancestry)	10.4452
53	NA07357	CEU (Utah residents with Northern and Western European ancestry)	6.18814
54	NA12234	CEU (Utah residents with Northern and Western European ancestry)	7.08588
55	NA18550	CHB (Han Chinese in Beijing, China)	7.78798
56	NA18570	CHB (Han Chinese in Beijing, China)	7.96559
57	NA18582	CHB (Han Chinese in Beijing, China)	8.63521

58	NA18944	JPT (Japanese in Tokyo, Japan)	5.58686
59	NA18973	JPT (Japanese in Tokyo, Japan)	4.97637
60	NA19005	JPT (Japanese in Tokyo, Japan)	7.20848
61	NA19238	YRI (Yoruba in Ibadan, Nigeria)	4.92286
62	NA19239	YRI (Yoruba in Ibadan, Nigeria)	4.67774
63	NA19240	YRI (Yoruba in Ibadan, Nigeria)	4.59382
64	NA19429	LWK (Luhya in Webuye, Kenya)	6.02545
65	NA19443	LWK (Luhya in Webuye, Kenya)	10.5005
66	NA19462	LWK (Luhya in Webuye, Kenya)	6.48215
67	NA19725	MXL (Mexican Ancestry in Los Angeles, California)	6.03405
68	NA19755	MXL (Mexican Ancestry in Los Angeles, California)	5.9912
69	NA19764	MXL (Mexican Ancestry in Los Angeles, California)	6.82051
70	NA19913	ASW (African Ancestry in Southwest US)	6.52439
71	NA20357	ASW (African Ancestry in Southwest US)	6.66108
72	NA20359	ASW (African Ancestry in Southwest US)	6.20712
73	NA20510	TSI (Toscani in Italia)	6.61414
74	NA20518	TSI (Toscani in Italia)	7.91557
75	NA20529	TSI (Toscani in Italia)	5.98378
76	NA20863	GIH (Gujarati Indian in Houston, TX)	7.7302
77	NA20867	GIH (Gujarati Indian in Houston, TX)	6.00576
78	NA20868	GIH (Gujarati Indian in Houston, TX)	6.68754

Supplemental Table S2: P-value for Chi-square statistical test of indel size distribution between the tools.

	**VarScan**	**Pindel**	**SAMtools**	**Dindel**	**GATK_HC**	**Platypus**
GATK_UG	0.99	0.69	0.8	0.27	0.35	0.99
VarScan		0.58	0.57	0.21	0.27	0.98
Pindel			0.71	0.98	0.99	0.88
SAMtools				0.96	0.98	0.58
Dindel					0.99	0.83
GATK_HC						0.80

Supplemental Figures:

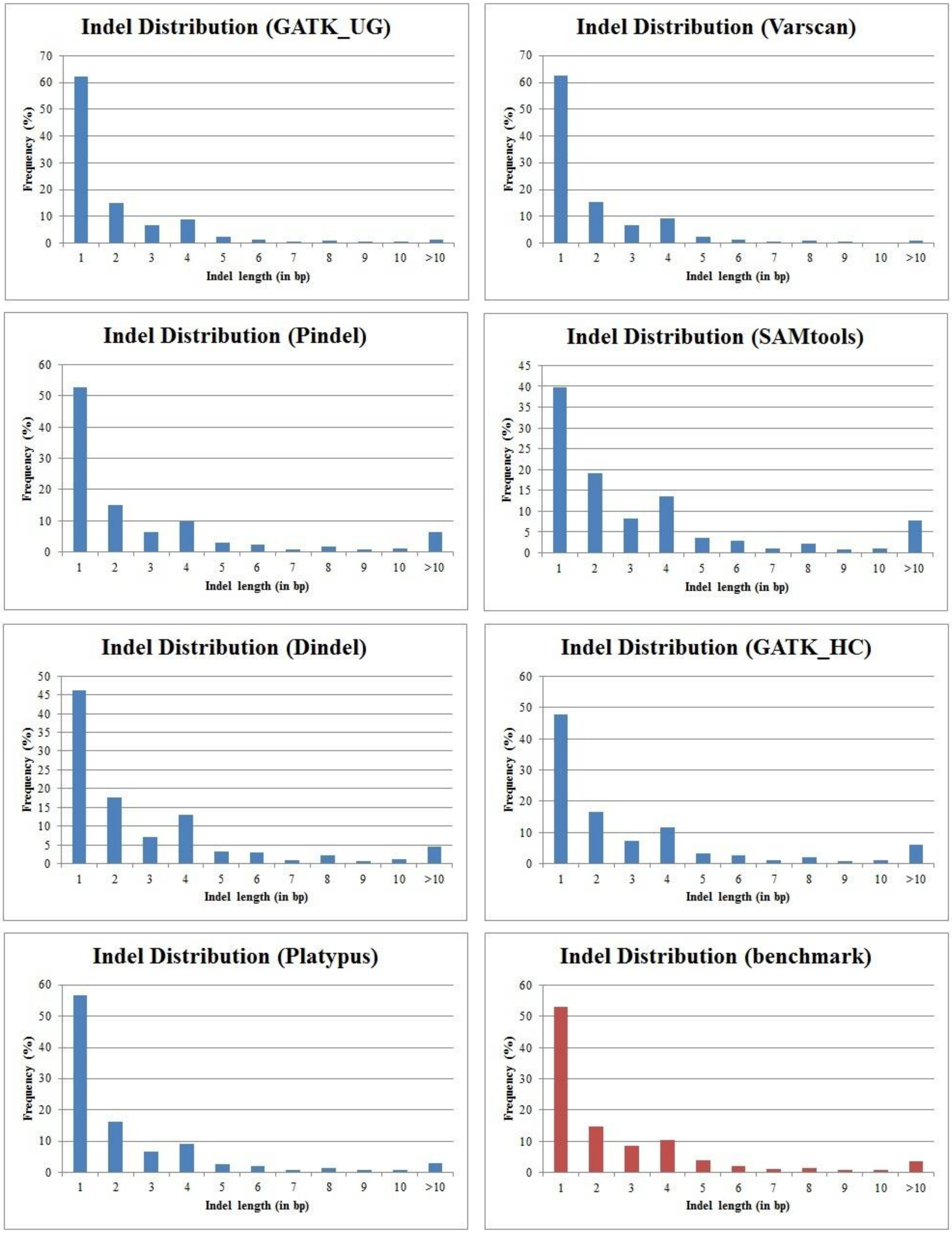

Supplemental Figure 1: Distribution of indels based on lengths (1 to 10 bp) for GATK_UG, Varscan, Pindel, SAMtools, Dindel, GATK_HC, Platypus and Benchmark dataset.

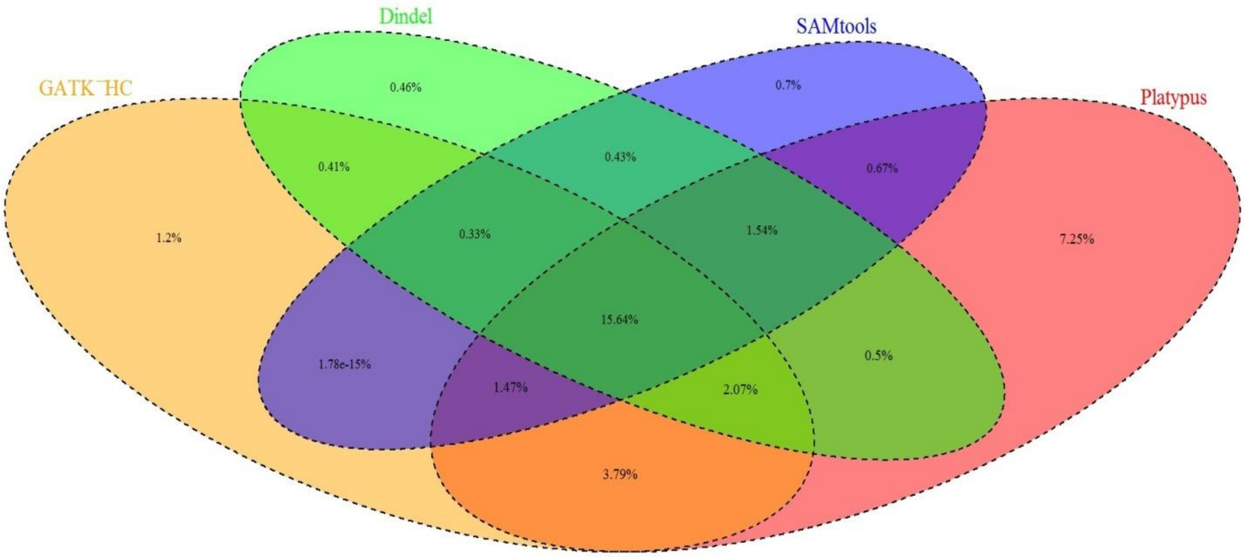

Supplemental Figure 2: Intra-tool comparison among GATK_HC, Dindel, SAMtools, and Platypus for percentage of their own indels called by others.

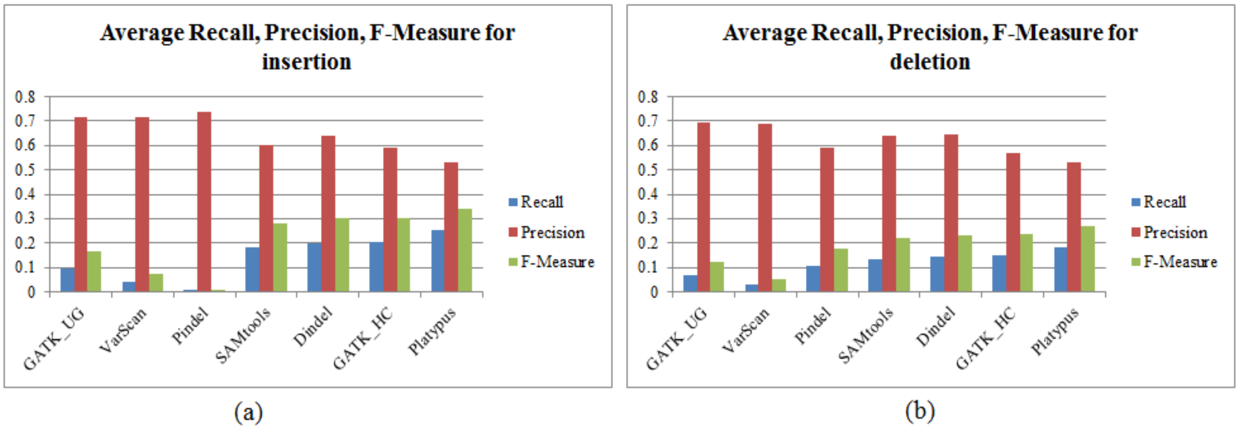

Supplemental Figure 3: Average Recall, Precision, and F-Measure of each tool for (a) insertion and (b) deletion.

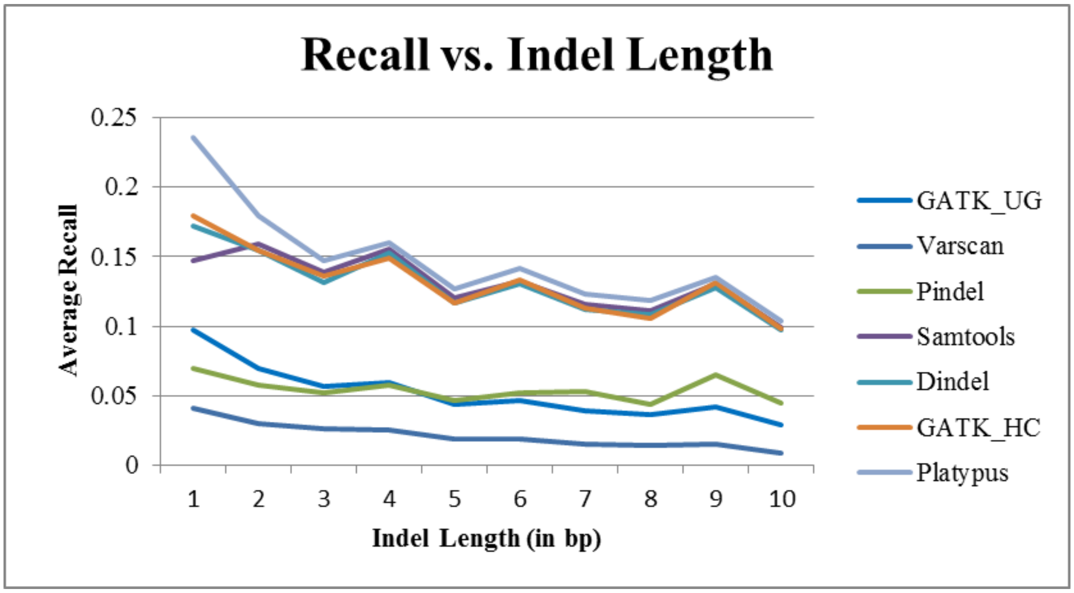

Supplemental Figure 4: Average recall for each tool for different lengths of indels.

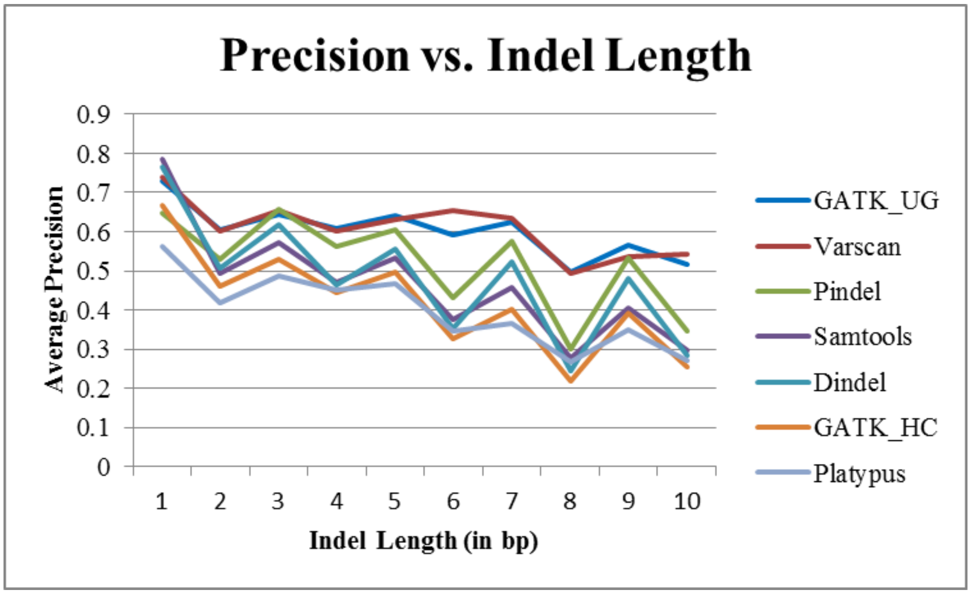

Supplemental Figure 5: Average precision for each tool for different lengths of indels.

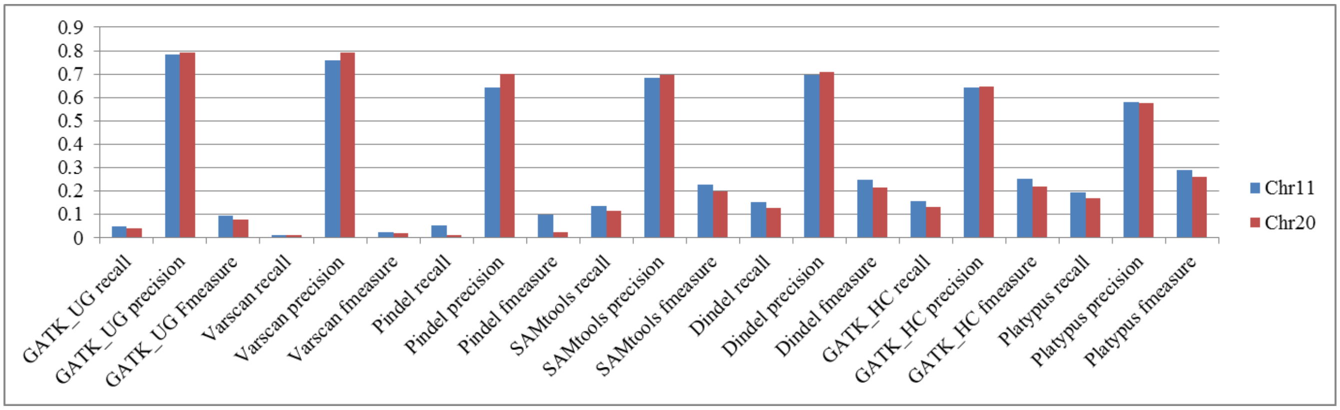

Supplemental Figure 6: Comparison between Chromosome 11 and Chromosome 20 for HG00157.

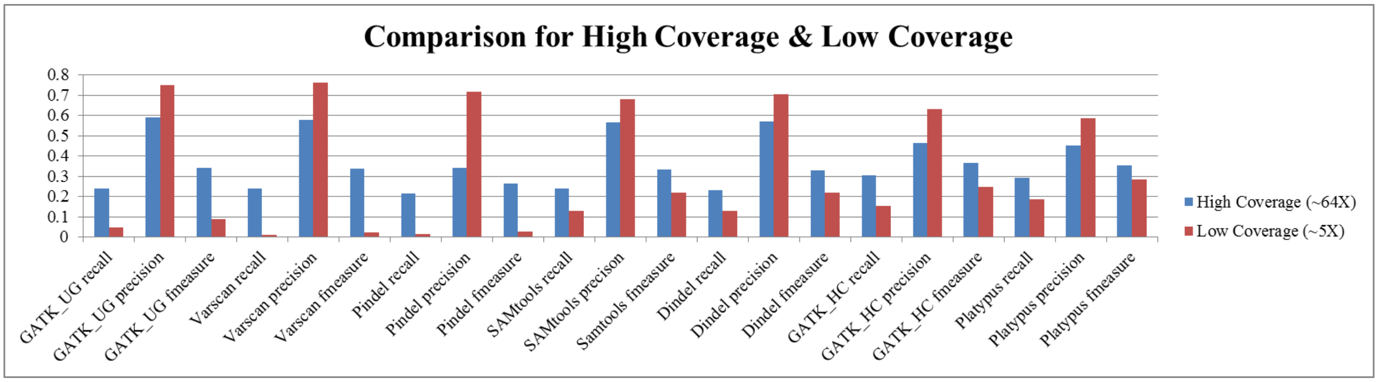

Supplemental Figure 7: Comparison between High (~64x) and Low (~5x) coverage samples for NA12878.
